# Supplementary material for: Integrated Analysis of the Lung Microbiome and Metabolome Reveals Associations Between Amino Acid Metabolism and Pulmonary Fibrosis in a Bleomycin-Induced Mouse Model
Source: Int J Mol Sci. 2026 Jun 30;27(13):5895. doi: 10.3390/ijms27135895 (PMC13362081; doi:10.3390/ijms27135895)
Supplement: Supplementary file 1 [file ijms-27-05895-s001.zip › result/4.MetDiffAnalysis/Box_diff/all/Com_2424_pos_Box_plot.pdf]

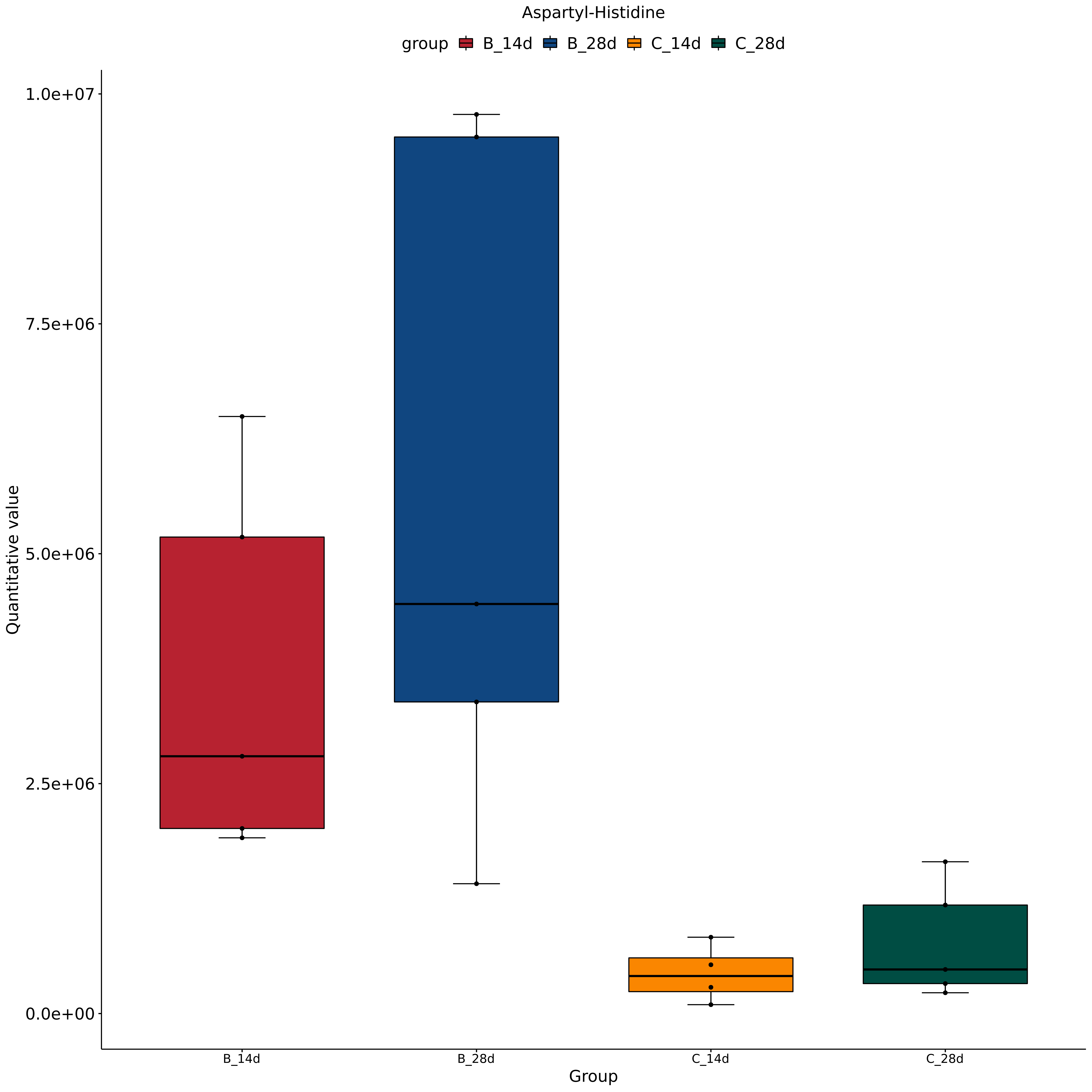

Aspartyl-Histidine
group B_14d B_28d C_14d C_28d
1.0e+07
7.5e+06
5.0e+06
2.5e+06
0.0e+00
Quantitative value
B_14d
B_28d
C_14d
C_28d
Group
